# GNSS Spoofing Detection and Mitigation Based on Maximum Likelihood Estimation

**DOI:** 10.3390/s17071532

**Published:** 2017-06-30

**Authors:** Fei Wang, Hong Li, Mingquan Lu

**Affiliations:** Department of Electronic Engineering, Tsinghua University, Beijing 100084, China; fei-wang11@mails.tsinghua.edu.cn (F.W.); lumq@tsinghua.edu.cn (M.L.)

**Keywords:** GNSS, maximum likelihood estimation, spoofing detection, spoofing mitigation, navigation solution recovery

## Abstract

Spoofing attacks are threatening the global navigation satellite system (GNSS). The maximum likelihood estimation (MLE)-based positioning technique is a direct positioning method originally developed for multipath rejection and weak signal processing. We find this method also has a potential ability for GNSS anti-spoofing since a spoofing attack that misleads the positioning and timing result will cause distortion to the MLE cost function. Based on the method, an estimation-cancellation approach is presented to detect spoofing attacks and recover the navigation solution. A statistic is derived for spoofing detection with the principle of the generalized likelihood ratio test (GLRT). Then, the MLE cost function is decomposed to further validate whether the navigation solution obtained by MLE-based positioning is formed by consistent signals. Both formulae and simulations are provided to evaluate the anti-spoofing performance. Experiments with recordings in real GNSS spoofing scenarios are also performed to validate the practicability of the approach. Results show that the method works even when the code phase differences between the spoofing and authentic signals are much less than one code chip, which can improve the availability of GNSS service greatly under spoofing attacks.

## 1. Introduction

The security of global navigation satellite system (GNSS) has caught more and more public attention because of the ever-increasing reliance on GNSS in our lives. GNSS receivers are vulnerable to jamming and spoofing attacks because the power of received GNSS signals is very low and the details of civilian signals are open to the public [1,2].

Spoofing signals can lead to wrong position, velocity and time (PVT) results. A victim receiver may output results preassigned by a spoofer so that the motion of the vehicle equipped with the receiver will be misled [3].

Many spoofing tests have been carried out over the last few years. The first GNSS spoofing experiment was presented by Warner and Johnston using a GS720 satellite simulator [4]. In the experiment, a spoofer was mounted on a truck, and the counterfeit signals were effective when the distance between the spoofer and the victim receiver did not exceed 30 feet. A portable Global Positioning System (GPS) civilian spoofer was developed by researchers at the University of Texas at Austin [3]. Spoofing threat was assessed with the device, and a successful spoofing experiment was carried out on a super yacht [5]. The researchers also did a successful spoofing test on phasor measurement units (PMU), which are employed in time synchronization of the power system [6]. It is obvious that the GNSS spoofing attack is not far from reality. Hence, efficient spoofing countermeasures need to be developed.

Many anti-spoofing techniques have been developed because of the importance of GNSS security. Some of them require additional hardware or changes to the interface specification. Techniques based on multiple antennas, absolute power measurements and inertial navigation system (INS) perform spoofing detection with measurements from additional sensors [7,8,9]. Cross-correlation techniques detect spoofing attack by performing cross-correlation of encrypted signals between secure and defended receivers [10]. Cryptographic methods, such as spread spectrum security code (SSSC) [11] and navigation message authentication (NMA) [12], attempt to prevent spoofing attacks by signal encryption or designing new GNSS signals, which are difficult to simulate. However, these methods are, to some extent, vulnerable to the replay attacks, in which the spoofer estimates, manipulates and replays the cryptographically-secured GNSS signals in real time [13,14].

The above-mentioned countermeasures require additional hardware, which increases the expense. Hence, their applications are limited. Other countermeasures are applied in single antenna receivers by detecting the abnormality and inconsistency of measurements. For example, signal quality monitoring (SQM) techniques detect the distortion of correlation results in the tracking stage [15]. Receiver autonomous integrity monitoring (RAIM) checks the consistency between pseudorange measurements of different satellites [16]. It fails when all channels are taken over by spoofing signals [17]. Moving antenna techniques detect and identify spoofing signals with the correlation between measurements of pairwise signals. It is desirable to be implemented in a moving single antenna handset [18,19,20]. The multiple tracking technique tracks all of the signals that are over the threshold in the acquisition stage and attempts to distinguish the authentic measurements from spoofing ones and recover the navigation solution [21]. It only works when the code phase difference between the authentic and spoofing signals is more than one code chip [22].

Most of the spoofing countermeasures mentioned above process signals separately, which limits their performance since the associated information between different signals is ignored. The maximum likelihood estimation (MLE)-based positioning technique is a direct positioning method originally developed for multipath rejection and weak signal processing [23]. In this paper, a jointly performed estimation-cancellation approach is proposed for spoofing detection and navigation solution recovery based on the technique. Firstly, a composite signal is reconstructed based on the estimated PVT parameters and subtracted from the original signal. Then, a test statistic is calculated with the residual signal to indicate whether there are evil signals such as spoofing and multipath signals. If evil signals are detected, a validation procedure is performed by inspecting the consistency of the signals to determine whether the evil signals are spoofing ones or not. Unlike the RAIM technique, the proposed method can detect spoofing attacks when all of the channels of the victim receivers are taken over by the spoofing signals, and it can even work when the code phase differences between the authentic and spoofing signals are much less than one code chip.

The outline of the paper is as follows. In Section 2, the GNSS baseband signal model is introduced. In Section 3, the maximum likelihood estimation for the GNSS PVT solution is reviewed. It is the foundation of the proposed method in the paper. In Section 4, an estimation-cancellation algorithm based on MLE is described, and a statistic for evil signal detection is derived based on GLRT. Furthermore, a metric is proposed for spoofing validation. The theoretical performance of the spoofing countermeasure is also derived in this section. In Section 5, both numerical and simulation results are presented to investigate the factors that affect the performance of the proposed method. In Section 6, the countermeasure is evaluated with the Texas Spoofing Test Battery (TEXBAT), which consists of recorded data in different real spoofing scenarios and has been widely used for GNSS anti-spoofing tests in the world. Finally, we conclude our work in Section 7.

## 2. Signal Model

The received GNSS signal can be transformed into a complex baseband signal. When no spoofing signals are present, the baseband signal can be expressed as follows [23,24]:

(1) $$x_{n}=\sum_{i=1}^{M}a_{i}D_{i}\left(nT_{s}-\tau_{i}\right)c_{i}\left(nT_{s}-\tau_{i}\right)e^{j(2\pi f_{i}nT_{s}+\theta_{i})}+w\left(nT_{s}\right),\;n=0,1,\cdots ,K-1.$$

Here,

- $F_{s}=1/T_{s}$ is the sampling frequency;
- *M* stands for the number of in-view satellites;
- *K* stands for the number of samples;
- $a_{i}$ is the amplitude of the *i*-th satellite signal;
- $D_{i}\left(t\right)$ is the navigation data of the *i*-th signal at time *t*;
- $c_{i}\left(t\right)$ is the pseudorandom code of the *i*-th signal at time *t*;
- $\tau_{i}$ is the propagation delay of the *i*-th signal;
- $f_{i}$ is the Doppler frequency offset of the *i*-th signal;
- $\theta_{i}$ is the carrier phase of the *i*-th signal;
- w(t) is complex zero-mean additive white Gaussian noise (AWGN) with variance $\sigma^{2}$.

Choosing *K* samples as observation data, Equation (1) can be rewritten as [23]:

(2) x=G(τ,f)a+w.

Here,

- $x={\left[x_{1},x_{2},\cdots ,x_{K}\right]}^{T}\in C^{K\times 1}$ is the measurement vector;
- G(τ,f) can be expressed as $\left[g_{1},\ldots ,g_{M}\right]$, being $g_{i}={\left[g_{1,i},\ldots ,g_{K,i}\right]}^{T}$, and $g_{k,i}=D_{i}\left(kT_{s}-\tau_{i}\right)c_{i}\left(kT_{s}-\tau_{i}\right)e^{j2\pi f_{d_{i}}kT_{s}}\;,\;k=1,2,\cdots ,K,i=1,2,\cdots ,M$;
- $\tau =[\tau_{1},\tau_{2},\cdots ,\tau_{M}]$, $f=[f_{1},f_{2},\cdots ,f_{M}]$. During the observation period, τ and f can be regarded as constants under static or low dynamic condition;
- $a={\left[a_{1}e^{j\theta_{1}},a_{2}e^{j\theta_{2}},\cdots ,a_{M}e^{j\theta_{M}}\right]}^{T}\in C^{M\times 1}$ represents complex amplitudes of the received signals;
- $w={\left[w\left(T_{s}\right),w\left(2T_{s}\right),\cdots ,w\left(KT_{s}\right)\right]}^{T}\in C^{K\times 1}$ is the AWGN vector, and each element has a variance $\sigma^{2}$ during the observation period.

## 3. Review on MLE-Based Positioning

Unlike a conventional GNSS receiver, which extracts measurements in several parallel tracking channels independently, the GNSS MLE-based positioning technique provides a way of jointly processing the signals of all in-view satellites. Taking advantage of the gain from the merging of signals, the method is robust against multipath and signal fading conditions [23]. In this section, we will review the MLE-based positioning, which is the foundation of the proposed method in the paper.

### 3.1. Maximum Likelihood Estimation

The probability density function (pdf) of the measurement vector x can be expressed as Equation (3) since the noise vector w is zero-mean AWGN with variance $\sigma^{2}$.

(3) $$\begin{array}{c} p\left(x;\tau ,f,a,\sigma^{2}\right)=\frac{1}{{\left(2\pi \sigma^{2}\right)}^{\frac{K}{2}}}exp[-\frac{||x-{\mathrm{Ga}||}^{2}}{2\sigma^{2}}]. \end{array}$$

Denote {τ,f} as r. The maximum likelihood (ML) estimates of the unknown parameters are given by [25]:

(4) $$\hat{r}=arg\max_{r}\left\{x^{H}G(r){\left[{G(r)}^{H}G\left(r\right)\right]}^{-1}{G(r)}^{H}x\right\}$$

(5) $$\hat{a}={\left[G{\left(\hat{r}\right)}^{H}G\left(\hat{r}\right)\right]}^{-1}G{\left(\hat{r}\right)}^{H}x.$$

(6) $${\hat{\sigma }}^{2}=\frac{1}{K}{\left[x-G\left(\hat{r}\right)\hat{a}\right]}^{H}\left[x-G\left(\hat{r}\right)\hat{a}\right].$$

(7) $$J\left(r;x\right)=x^{H}G(r){\left[{G(r)}^{H}G\left(r\right)\right]}^{-1}{G(r)}^{H}x$$

We call *J* the cost function of MLE in the rest of the paper. The ML estimates of the parameters can be obtained by maximizing the cost function.

### 3.2. Position Estimation

The MLE cost function is determined by the synchronization parameters τ and f. These parameters can be calculated with the user PVT parameters. The elements in τ and f are given by [26]:

(8) $$\tau_{i}=\frac{1}{c}∥u^{\left(i\right)}-u∥+t_{u}-t^{\left(i\right)}+e^{\left(i\right)},$$

(9) $$f_{i}=-\frac{f_{T}}{c}\left[\left(v^{\left(i\right)}-v\right)1^{\left(i\right)}+{\dot{t}}_{u}-{\dot{t}}^{\left(i\right)}\right].$$

Here,

- *c* is the speed of light;
- $u^{\left(i\right)}$ is the coordinates of the *i*-th satellite in Earth-centered earth-fixed (ECEF) coordinate system;
- $u={\left[x_{u},y_{u},z_{u}\right]}^{T}$ is the coordinates of the user in ECEF coordinate system;
- $t_{u}$ is the offset of the user clock from system time;
- $t^{\left(i\right)}$ is the offset of the *i*-th satellite clock from system time;
- $e^{\left(i\right)}$ denotes ionospheric and tropospheric delays and errors induced by relativistic effects; these errors can be eliminated by models or differential techniques;
- $f_{T}$ is the frequency of the transmitted satellite signals;
- $v^{\left(i\right)}$ is the velocity of the *i*-th satellite in ECEF coordinate system;
- $v={\left[x_{v},y_{v},z_{v}\right]}^{T}$ is the velocity of the user in ECEF coordinate system;
- $1^{\left(i\right)}$ is the unit vector pointing along the line of sight from the user to the *i*-th satellite and can be expressed as Equation (10);
- ${\dot{t}}_{u}$ is the drift rate of the user clock relative to system time;
- ${\dot{t}}^{\left(i\right)}$ is the drift rate of the *i*-th satellite clock relative to system time.

(10) $$1^{\left(i\right)}=-\frac{u^{\left(i\right)}-u}{∥u^{\left(i\right)}-u∥}.$$

It can be seen that τ and f are determined by the user PVT parameters ρ = [u, $t_{u}$, v, ${\dot{t}}_{u}]$. When at least four satellites are in view, ρ can also be resolved by τ and f. Hence, the ML estimates of the user PVT parameters can be obtained by maximizing *J* based on the invariance principle of MLE [23].

## 4. MLE-Based GNSS Anti-Spoofing Method

Since the MLE cost function is directly connected with the PVT parameters, spoofing signals that mislead the PVT results will cause distortion to the MLE cost function. Hence, the MLE-based positioning technique has a potential ability for spoofing detection. In this section, based on the technique, an estimation-cancellation algorithm is proposed, and a test statistic is derived for the detection of evil signals. Then, a validation method is proposed based on the decomposition of MLE cost function to determine whether the evil signals are spoofing ones or not. Finally, the overall implementation architecture is provided at the end of the section.

### 4.1. Model of Spoofing Signal

When there is a spoofing attack, the baseband signal can be expressed as:

(11) $$x=G\left(\rho_{a}\right)a_{a}+G\left(\rho_{s}\right)a_{s}+w,$$

where $\rho_{a}$ and $a_{a}$ are the PVT and amplitude parameters of the authentic signals and $\rho_{s}$ and $a_{s}$ are the corresponding parameters of the spoofing ones. Since a spoofer aims to mislead the PVT results of the victim receiver, $\rho_{s}$ is different from $\rho_{a}$. Consequently, the authentic and spoofing signals will form different peaks in the cost function.

**Figure 1:**
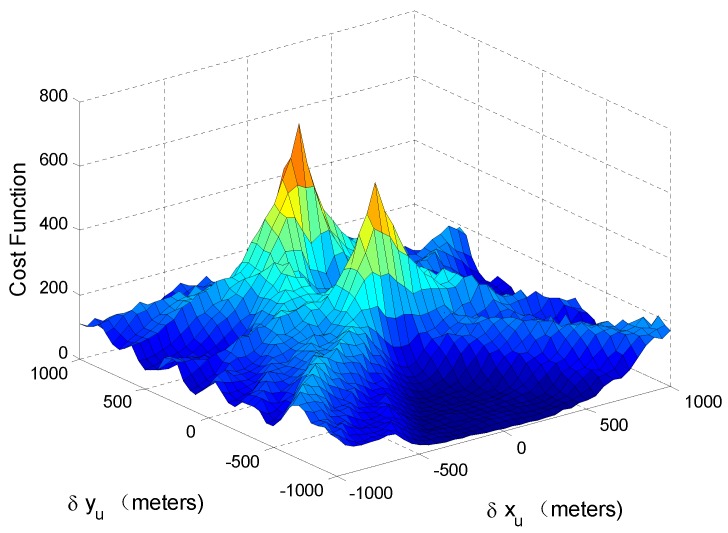
MLE cost function in a scenario with eight authentic signals and eight spoofing ones.

In order to view the MLE cost function under a spoofing attack intuitively, it is assumed that only $x_{u}$ and $y_{u}$ are unknown, and the search space is limited to $\left[x_{u},y_{u}\right]$. The two-dimensional cost function *J* with eight authentic signals and eight spoofing ones is shown in Figure 1. The center of the search space corresponds to $\rho_{a}$ or $\left[\delta x_{u},\delta y_{u}\right]=\left[0,0\right]$. The position offset induced by the spoofing signals is $\left[\delta x_{u},\delta y_{u}\right]=\left[0,600\right]$ m, and the other parameters in $\rho_{a}$ and $\rho_{s}$ are the same. The $C/N_{0}$ of each authentic signal is 45 dB-Hz, and the power of a spoofing signal is 0.8 dB higher than that of the corresponding authentic one. It can be seen that when $\left[\delta x_{u},\delta y_{u}\right]=\left[0,600\right]$ m, the cost function is maximized, and when $\left[\delta x_{u},\delta y_{u}\right]=\left[0,0\right]$, another peak, which is slightly lower than the maximum value of the cost function, can be found. If the MLE-based positioning is employed, since the power of the spoofing signals is higher than that of the authentic ones, the peak at $\rho_{s}$ will be the highest, and the ML estimate of ρ will be equal to $\rho_{s}$. That is to say, when the conventional MLE-based positioning method is used, the spoofing attack can still mislead the PVT result.

### 4.2. Estimation-Cancellation Approach

After obtaining the ML estimate of ρ, which is denoted as ${\hat{\rho }}_{1}$, the composite signal can be reconstructed as follows:

(12) $$\hat{x}=G\left({\hat{\rho }}_{1}\right){\hat{a}}_{1}=G\left({\hat{\rho }}_{1}\right){\left[G{\left({\hat{\rho }}_{1}\right)}^{H}G\left({\hat{\rho }}_{1}\right)\right]}^{-1}G{\left({\hat{\rho }}_{1}\right)}^{H}x$$

Then, we subtract the reconstructed signal from the original signal. The residual signal plus noise is given by:

(13) $$y=x-\hat{x}$$

When there is no spoofing signal, ${\hat{\rho }}_{1}$ is equal to $\rho_{a}$, and $\hat{x}$ is the estimation of the sum of the authentic signals. Thus, y mainly consists of noise. When spoofing signals are present, y will consist of residual signals and noise because $\rho_{s}$ is different from $\rho_{a}$. Based on the above analysis, y can be expressed as follows:

(14) $$\begin{array}{cc} H_{0}:y & =w\\ H_{1}:y & =G\left(\rho_{s}\right)a_{s}+G\left(\rho_{a}\right)a_{a}-G\left({\hat{\rho }}_{1}\right){\hat{a}}_{1}+w \end{array}$$

where $H_{0}$ denotes that spoofing signals are absent and $H_{1}$ denotes that spoofing signals are present. A spoofing attack can be detected by detecting signal components in y, and we call it the estimation-cancellation approach.

**Figure 2:**
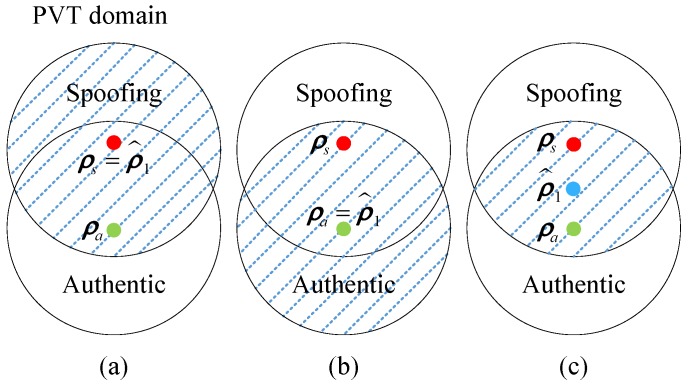
Three different cases when spoofing signals are present. The circles represent the PVT domains in which spoofing signals or authentic signals are present. The highest peak of the MLE cost function at ${\hat{\rho }}_{1}$ is superimposed by the signal components in the area emphasized by the dashed lines. (**a**) ${\hat{\rho }}_{1}$ is equal to $\rho_{s}$; (**b**) ${\hat{\rho }}_{1}$ is equal to $\rho_{a}$; (**c**) ${\hat{\rho }}_{1}$ is neither equal to $\rho_{s}$ nor to $\rho_{a}$.

The alternative hypothesis $H_{1}$ consists of three cases, which are illustrated in Figure 2. Figure 2 is an abstract expression of the PVT domain. Spoofing and authentic signal components form peaks at different positions of the domain. Figure 1 shows that the spoofing and authentic signal components are distributed around $\rho_{s}$ and $\rho_{a}$, and the corresponding PVT domains are represented with circles centered at $\rho_{s}$ and $\rho_{a}$ in Figure 2, respectively. The highest peak of the MLE cost function at ${\hat{\rho }}_{1}$ is superimposed by the signal components in the area emphasized by dashed lines.

Figure 2a represents the first case. The total power of the spoofing signals is higher than that of the authentic ones. Therefore, the MLE cost function is maximized at $\rho_{s}$ and the residual signal y mainly consists of authentic signals and noise. The spoofing scenario shown in Figure 1 is a good illustration for this case. It should be noted that when the designated falsified PVT parameter $\rho_{s}$ is close to $\rho_{a}$, parts of authentic signals will also contribute to the peak, which corresponds to the overlapped part of the circles in Figure 2a.

Figure 2b represents the second case, which is opposite the first case. It happens when the total power of the spoofing signals is lower than that of the authentic ones. Consequently, the MLE cost function is maximized at $\rho_{a}$, and the residual signal y mainly consists of spoofing signals and noise. In this case, the authentic PVT results can be obtained with the MLE-based positioning method directly.

In particular, when $\rho_{s}$ is far away from $\rho_{a}$, the two circles representing spoofing and authentic signal components will be separate in Figure 2a,b.

Figure 2c represents the third case. ${\hat{\rho }}_{1}$ is different from both $\rho_{s}$ and $\rho_{a}$. Thus, the residual signal consists of residual authentic signals, spoofing signals and noise.

We will discuss the three cases respectively in the rest of the subsection. As for the first case, we will first consider the limiting case in which the two circles in Figure 2a are separate. All of the authentic signals will be reserved in y, and the hypothesis test in Equation (14) can be rewritten as follows:

(15) $$\begin{array}{cc} H_{0}:y & =w\\ H_{1}:y & =G\left(\rho_{a}\right)a_{a}+w \end{array}$$

Then, we perform the MLE-based positioning again with y. The ML estimates of $\tau_{a}$, $f_{a}$, $a_{a}$ and $\sigma^{2}$ under $H_{1}$ can be obtained with Equations (4)–(6) by replacing x with y, and they are denoted as ${\hat{\tau }}_{2}$, ${\hat{f}}_{2}$, ${\hat{a}}_{2}$ and ${\hat{\sigma }}_{2}^{2}$, respectively. The only unknown parameter under $H_{0}$ is the variance of noise, which is denoted as $\sigma_{0}^{2}$, and its ML estimate is given by:

(16) $${\hat{\sigma_{0}}}^{2}=\frac{1}{K}y^{H}y$$

Then, with the ML estimates mentioned above, the generalized likelihood ratio test (GLRT) statistic can be derived as:

(17) $$\begin{array}{cc} L_{G}\left(y\right) & =\frac{\max_{\tau ,f,a,\sigma^{2}}p_{1}\left(y;\tau ,f,a,\sigma^{2}\right)}{\max_{\sigma^{2}}p_{0}\left(y;\sigma^{2}\right)}\\ & ={\left(\frac{{\hat{\sigma }}_{0}^{2}}{{\hat{\sigma }}_{2}^{2}}\right)}^{\frac{K}{2}}={\left\{\frac{y^{H}y}{{\left[y-G\left({\hat{\rho }}_{2}\right){\hat{a}}_{2}\right]}^{H}\left[y-G\left({\hat{\rho }}_{2}\right){\hat{a}}_{2}\right]}\right\}}^{\frac{K}{2}} \end{array}$$

where $p_{0}\left(y;\sigma^{2}\right)$ and $p_{1}\left(y;\tau ,f,a,\sigma^{2}\right)$ denote the likelihood functions under $H_{0}$ and $H_{1}$, respectively. ${\hat{\rho }}_{2}$ is the ML estimate of the PVT result corresponding to the residual signal y. Using the monotonicity of the function $f\left(x\right)=a\left(1-1/x^{c}\right),x,a,c>0$, we have:

(18) $$\begin{array}{cc} T(y) & =2K[1-\frac{1}{L_{G}{\left(y\right)}^{\frac{2}{K}}}]\\ & =2\frac{y^{H}y-{\left[y-G\left({\hat{\rho }}_{2}\right){\hat{a}}_{2}\right]}^{H}\left[y-G\left({\hat{\rho }}_{2}\right){\hat{a}}_{2}\right]}{y^{H}y/K}\\ & =\frac{{\hat{a}}_{2}^{H}G{\left({\hat{\rho }}_{2}\right)}^{H}G\left({\hat{\rho }}_{2}\right){\hat{a}}_{2}}{{\hat{\sigma }}_{0}^{2}/2}\\ & =\frac{y^{H}G\left({\hat{\rho }}_{2}\right){\left[G{\left({\hat{\rho }}_{2}\right)}^{H}G\left({\hat{\rho }}_{2}\right)\right]}^{-1}G{\left({\hat{\rho }}_{2}\right)}^{H}y}{{\hat{\sigma }}_{0}^{2}/2}\\ & =\frac{\max_{\rho_{2}}J\left(\rho_{2};y\right)}{{\hat{\sigma }}_{0}^{2}/2}>\gamma \end{array}$$

**Figure 3:**
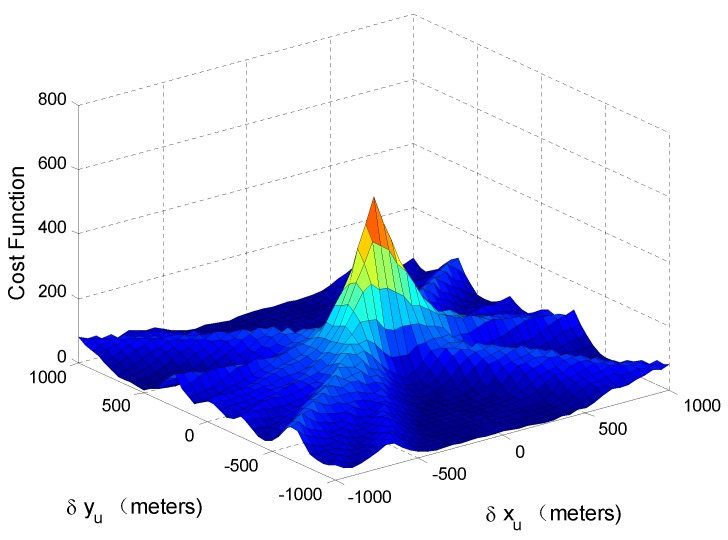
MLE cost function after estimation-cancellation.

The cost function $J(\rho_{2};y)$ after estimation-cancellation is shown in Figure 3. All of the simulation parameters are the same as those in Figure 1. It can be seen that when $\left[\delta x_{u},\delta y_{u}\right]=\left[0,0\right]$, the cost function is maximized. Figure 3 shows that $\rho_{a}$ can be obtained by maximizing $J(\rho_{2};y)$ in the first case.

T(y) follows *F* and non-central *F* distributions under $H_{0}$ and $H_{1}$, respectively [25]. Since the estimation are performed with a large number of samples considering the sampling frequency and processing time, the ML estimate ${\hat{\sigma }}_{0}^{2}$ is very close to its true value $\sigma^{2}$. Therefore, T(y) follows central $\chi^{2}$ distribution under $H_{0}$ and non-central $\chi^{2}$ distribution under $H_{1}$.

(19) $$T\left(y\right)∼\left\{\begin{array}{c} \chi_{2M}^{2},\;under\;H_{0}\\ \chi_{2M}^{\prime 2}\left(\lambda \right),\;under\;H_{1} \end{array}\right.$$

The non-central parameter is given by [25]:

(20) $$\lambda =\frac{a^{H}G{\left(\rho_{2}\right)}^{H}G\left(\rho_{2}\right)a}{\sigma^{2}/2}$$

Thus, the false alarm and detection probabilities are given by [27]:

(21) $$P_{fa}\left(\gamma ,M\right)=\exp\left\{-\frac{\gamma }{2}\right\}\sum_{i=0}^{M-1}\frac{1}{i!}{\left(\frac{\gamma }{2}\right)}^{i}$$

(22) $$P_{d}\left(\gamma ,M\right)=Q_{M}\left(\sqrt{\lambda },\sqrt{\gamma }\right)$$

where γ is the threshold which can be determined by $P_{fa}$. $Q_{M}\left(a,b\right)$ is the generalized Marcum Q-function [28].

Further, by using the properties of cross-correlation of different satellites’ pseudorange codes, we have [29]:

(23) $$G{\left({\hat{\rho }}_{2}\right)}^{H}G\left({\hat{\rho }}_{2}\right)\approx KI_{M\times M}$$

Substituting Equation (23) to Equations (18) and (20), we have:

(24) $$\begin{array}{cc} T(y) & =\frac{y^{H}G\left({\hat{\rho }}_{2}\right){\left[G{\left({\hat{\rho }}_{2}\right)}^{H}G\left({\hat{\rho }}_{2}\right)\right]}^{-1}G{\left({\hat{\rho }}_{2}\right)}^{H}y}{{\hat{\sigma }}_{0}^{2}/2}\\ & \approx \frac{\frac{1}{K}y^{H}G\left({\hat{\rho }}_{2}\right)G{\left({\hat{\rho }}_{2}\right)}^{H}y}{{\hat{\sigma }}_{0}^{2}/2}\\ & =\frac{2}{K{\hat{\sigma }}_{0}^{2}}\sum_{i=1}^{M}\left|\right|g_{i}{\left({\hat{\rho }}_{2}\right)}^{H}y{\left|\right|}^{2} \end{array}$$

(25) $$\lambda \approx K\sum_{i=1}^{M}\frac{a_{i}^{2}}{\sigma^{2}/2}=2KT_{s}\sum_{i=1}^{M}{\left(C/N_{0}\right)}_{i}$$

where ${\left(C/N_{0}\right)}_{i}$ denotes the carrier to noise ratio of the *i*-th satellite in Hz. The equations show that the test statistic T(y) can be viewed as the noncoherent sum of different signals’ integration results.

The above analysis is based on the assumption that $\rho_{s}$ and $\rho_{a}$ are separate, and all of the authentic signals are reserved in the residual signal. However, when the peaks of the MLE cost function formed by spoofing and authentic signals are close, parts of the authentic signals will be subtracted in the cancellation procedure. Consequently, the signal components in Equation (15) under $H_{1}$ will be fewer. Thus, the detection performance, which is given by Equation (22), can be viewed as the upper bound. The detection performance will improve when the distance between $\rho_{s}$ and $\rho_{a}$ increases, and it will approach the upper bound after the peaks formed by the spoofing and authentic signals are separated completely.

The detection performance of the second case is very similar to that of the first one. It can also be evaluated with Equations (21), (22) and (25). Different from the first case, the ${\left(C/N_{0}\right)}_{i}$ in Equation (25) represents the carrier to noise ratio of the *i*-th spoofing signal.

As for the third case, as long as spoofing signals are present and mislead the PVT results of a receiver (i.e., $\rho_{s}\neq \rho_{a}$), y will include residual signal components. Therefore, the test statistic T(y) will exceed the threshold and can also be used for spoofing detection in this case. However, the method may fail to detect the spoofing attacks when $\rho_{s}$ is very close to $\rho_{a}$. Therefore, it is not fit for the applications that require high-precision PVT results. It should be noted that there is no exact expression for the detection performance of the third case. Nevertheless, since most of the spoofing and authentic signals that are superimposed in the PVT domain are eliminated, Equation (22) can still be viewed as the upper bound of the detection performance.

### 4.3. Spoofing Validation and Recovery of Navigation Solution

It should be noted that when there are severe multipath signals, T(y) can also exceed the threshold. However, the multipath signals are hardly consistent with each other since they are generated by different reflectors around a receiver. Different from the multipath signals, the spoofing ones may have the collaboration since they are generated sophisticatedly by a spoofer. Based on the difference, the following discrimination method is presented to reduce the false alarms induced by multipath signals and to validate the navigation solution.

After evil signals are detected, we calculate the following statistics.

(26) $$\begin{array}{c} T_{1i}\left(x\right)=\frac{2}{K{\hat{\sigma }}_{0}^{2}}\left|\right|g_{i}{\left({\hat{\rho }}_{1}\right)}^{H}x{\left|\right|}^{2}\;,\;i=1,2,\cdots ,M\\ T_{2i}\left(y\right)=\frac{2}{K{\hat{\sigma }}_{0}^{2}}\left|\right|g_{i}{\left({\hat{\rho }}_{2}\right)}^{H}y{\left|\right|}^{2}\;,\;i=1,2,\cdots ,M \end{array}$$

Then, count the statistics $T_{1i}\left(x\right)$ and $T_{2i}\left(y\right)$ that are over a threshold $\gamma^{\prime }$, respectively. Since the expressions of $T_{1i}\left(x\right)$ and $T_{2i}\left(y\right)$ are the same as the noncoherent integration result of a single satellite, $\gamma^{\prime }$ can be given by [26]:

(27) $$\gamma^{\prime }=-2ln\left(P_{fa}\right)$$

Denote the numbers of $T_{1i}\left(x\right)$ and $T_{2i}\left(y\right)$ that are over $\gamma^{\prime }$ as $r_{1}$ and $r_{2}$, respectively. If $r_{1}$ and $r_{2}$ are larger than four simultaneously, two groups of consistent signals are found in x, and the detected evil signals can be determined as consistent spoofing signals. The tenet of the method is similar to RAIM, which is based on the self-consistency of pseudorange measurements. In RAIM, the consistency can be determined when more than four measurements are used [26], and in the proposed validation method, signals can be judged as self-consistent only if the peak of the MLE cost function is superimposed by more than four signals.

It should be noted that if the multipath signals form consistent measurements, they cannot be distinguished from the spoofing signals. However, in this case, the multipath signals are very similar to the spoofing ones and may mislead the PVT results in the same way as the spoofing ones. Consequently, they can also be categorized as spoofing signals. The spoofing validation probability can be expressed as:

(28) $$\begin{array}{cc} P_{V} & =P_{V1}\cdot P_{V2}\\ P_{Vk} & =p(\;r_{k}>4\;),k=1,2 \end{array}$$

where p(E) denotes the probability of event *E*.

Particularly, when all of the signals’ $C/N_{0}$ are the same, $P_{Vk}$ has the following simplified form:

(29) PVk=∑n=5MMnPdkn(1-Pdk)M-n=1-B(4;M,Pdk)

where B(k;N,p) denotes the cumulative binomial distribution function and $P_{dk}$ denotes the detection probability of the single signal in the *k*-th MLE positioning module, which can be calculated with Equations (22) and (25) by setting *M* to one.

In order to see when a spoofing detection or a spoofing validation can be performed successfully, we further classify the cases in Figure 2 into nine different scenarios based on the numbers of spoofing and authentic signals in x and y. Since a peak of the MLE cost function is formed by consistent signals, spoofing signals with inconsistent measurements will not contribute to the peak at $\rho_{s}$, and their effects will be eliminated by the MLE-based method directly. Therefore, only consistent spoofing signals are considered. The classifications are given in Table 1. **spf** and **auth** denote the numbers of the spoofing and authentic signals, respectively.

Case 1 consists of Scenarios 1, 2 and 3. In these scenarios, consistent spoofing signals are more than four, and the total power of them is higher than that of the authentic ones. Therefore, ${\hat{\rho }}_{1}$ equals $\rho_{s}$, and only authentic signals are reserved in y.

In Scenario 1, $\rho_{s}$ and $\rho_{a}$ are separate. Therefore, the number of authentic signals is more than four in y, and both $r_{1}$ and $r_{2}$ are larger than four. Consequently, both spoofing detection and validation are successful.

In Scenario 2, $\rho_{s}$ and $\rho_{a}$ are close. Therefore, parts of the authentic signals are eliminated in the cancellation procedure, and the residual authentic signals are fewer than four. Consequently, $r_{2}$ is no more than four, and the spoofing validation fails. However, when the PVT bias induced by the spoofing signals becomes larger (i.e., $\rho_{s}$ is farther from $\rho_{a}$), the peaks of the MLE cost function formed by the spoofing and authentic signals will separate, and this scenario will turn into Scenario 1.

In Scenario 3, the number of the authentic signals is fewer than four due to the blockage in different traffic conditions. Consequently, $r_{2}$ is no more than four, and the spoofing validation also fails.

Case 2 consists of Scenarios 4, 5 and 6. In these scenarios, authentic signals are more than four, and the total power of them is higher than that of the spoofing ones. Therefore, ${\hat{\rho }}_{1}$ equals $\rho_{a}$, and only spoofing signals are reserved in y.

Scenarios 4, 5 and 6 are symmetric to Scenarios 1, 2 and 3, respectively. The detection and validation results are the same as those in Case 1.

Case 3 consists of Scenarios 7, 8 and 9. In these scenarios, the peak at ${\hat{\rho }}_{1}$ is formed by parts of spoofing signals and parts of authentic ones. Since at most four authentic and spoofing signals can be superimposed in the search domain, $r_{1}$ is no more than four, and the spoofing validation fails.

Even though only Scenarios 1 and 4 can be validated successfully. The validation is very meaningful since these two scenarios include the intermediate and sophisticated spoofing attacks mentioned in [3], which are very covert since they mislead the PVT results while maintaining the tracking state of a GNSS receiver.

In such spoofing attacks, the spoofer can extract the ephemeris and estimate the position and motion of a victim receiver in real time, based on which spoofing signals, which are consistent with the observed constellation, can be generated.

At the initial stage of the spoofing attacks, spoofing signals try to align their code phases to those of the corresponding authentic ones; consequently, $\rho_{s}\approx \rho_{a}$. Then, the spoofing signals increase their powers and take control of the delay lock loops of the victim receiver. Finally, they drag the code phases away from the true values, and $\rho_{s}$ will not be approximately equal to $\rho_{a}$ any longer.

When the code phases of the spoofing and authentic signals are perfectly aligned, the proposed method cannot detect the attack. However, since the aim of a spoofing attack is to mislead the PVT results, the bias between $\rho_{s}$ and $\rho_{a}$ will not maintain as zero, and it will increase gradually. Afterwards, the spoofing attack can be detected, and the navigation solution can be recovered.

The spoofing detection, validation and navigation recovery of such spoofing attacks in real tests will be provided in Section 6.

### 4.4. Implementation Architecture

**Figure 4:**
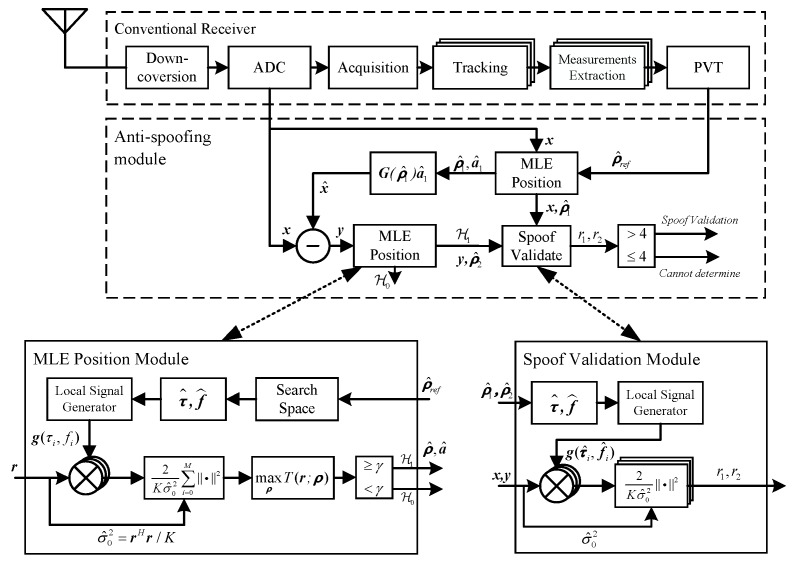
Implementation architecture.

The implementation architecture of the proposed method is shown in Figure 4. An anti-spoofing module can be embedded into a conventional receiver. It consists of two MLE position modules, one signal reconstruction module and one spoofing validation module. The first MLE position module is used to obtain the refined PVT result ${\hat{\rho }}_{1}$ and the corresponding amplitude estimation ${\hat{a}}_{1}$. After that, the reconstructed signal $\hat{x}$ is subtracted from the original baseband signal x, and the residual signal y is obtained. Then, y is input to the second MLE position module. When the test statistic is over the threshold, evil signals are detected. Then, x, y, ${\hat{\rho }}_{1}$ and ${\hat{\rho }}_{2}$ are input to the spoofing validation module. If both $r_{1}$ and $r_{2}$ are larger than four, two groups of consistent signals are detected. Thus, the evil signals in x are validated to be spoofing ones, and one of ${\hat{\rho }}_{1}$ and ${\hat{\rho }}_{2}$ corresponds to the authentic navigation solution.

## 5. Simulation Results

In the previous section, the MLE-based anti-spoofing countermeasure is presented, and the theoretical performance is derived. In this section, theoretical and simulation detection probabilities and the receiver operating characteristic (ROC) curves are provided to validate the analytical results. Spoofing validation probabilities are also evaluated.

GPS L1 C/A signals are employed in the simulations. The common simulation parameters are given as follows. The coherent integration time is 1 ms; the signals are sampled at a rate of 5 MHz; and the false alarm probability is set to $10^{-6}$.

**Figure 5:**
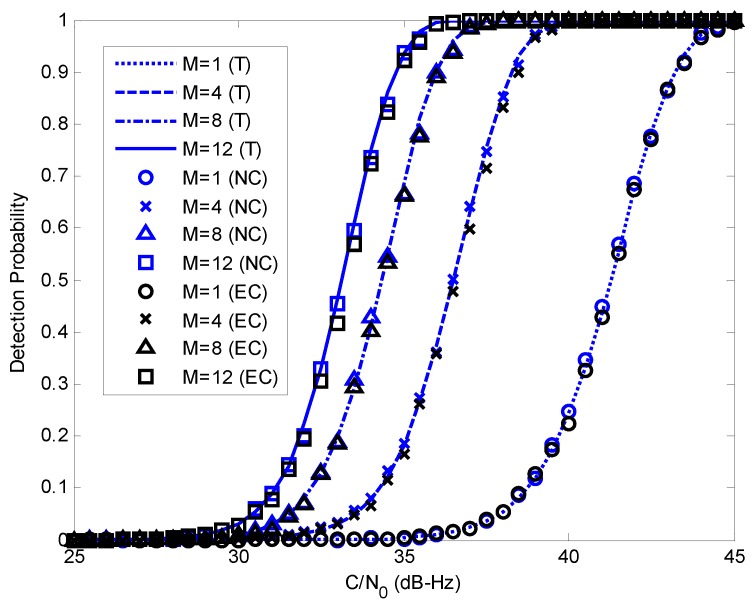
Detection probabilities versus $C/N_{0}$ for different numbers of satellites and a fixed false alarm probability $P_{fa}=10^{-6}$. $C/N_{0}$ of all of the spoofing signals are 50 dB-Hz. ’T’ represents the theoretical results. ’NC’ represents corresponding noncoherent integration results. ’EC’ represents the estimation-cancellation results.

In Figure 5, detection probabilities for different numbers of satellites and a fixed false alarm probability are compared. For simplicity and clarity, all of the $C/N_{0}$ of the authentic signals are equal, and those of the spoofing signals are set to 50 dB-Hz. The code phase differences between authentic, and spoofing signals for different PRNs are all set to 600 m. Thus, the correlation peaks of authentic and spoofing signals are separate. Theoretical results are shown with different types of lines (’T’). Estimation-cancellation results are shown with black markers (’EC’). Corresponding noncoherent integration results of *M* satellites are also provided for comparison, shown with blue markers (’NC’). Figure 5 demonstrates that the noncoherent integration results are almost the same as the theoretical results, but the estimation-cancellation results are slightly lower than the theoretical curves due to the small power loss of the authentic signals in the cancellation procedure. It also shows that the detection performance is better when more signals are processed simultaneously.

**Figure 6:**
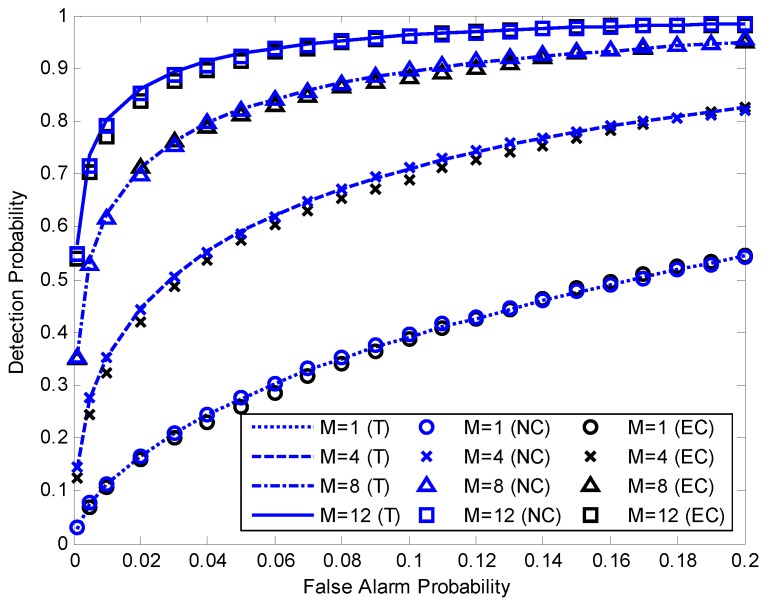
ROC curves for different numbers of satellites. $C/N_{0}$ of all of the authentic signals are 31 dB-Hz, and those of the spoofing signals are 50 dB-Hz. ’T’ represents the theoretical results. ’NC’ represents corresponding noncoherent integration results. ’EC’ represents the estimation-cancellation results.

Figure 6 shows the ROC curves for different numbers of satellites. All of the $C/N_{0}$ of the authentic signals are set to 31 dB-Hz, and the $C/N_{0}$ of the spoofing signals and the code phase differences are the same as those in Figure 5. Similar to Figure 5, the noncoherent integration results match the theoretical results well, and the detection probabilities of the estimation-cancellation algorithm are slightly lower than the theoretical results. Figure 5 and Figure 6 demonstrate that the detection performance can be evaluated with Equations (21) and (22) when the code phases of spoofing and authentic signals are separate.

**Figure 7:**
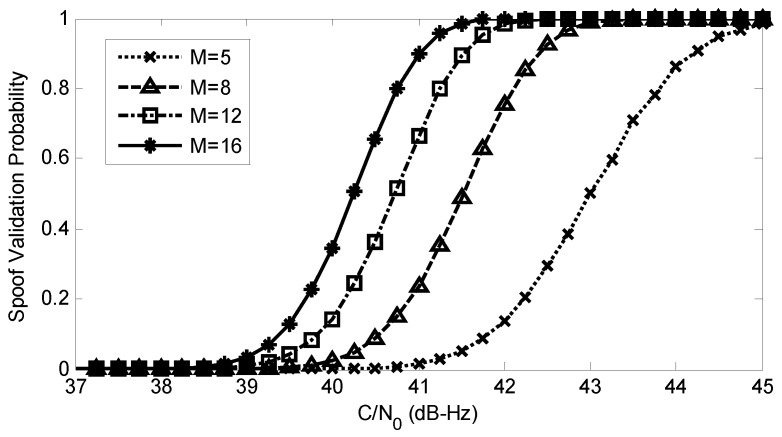
Spoofing validation probabilities versus $C/N_{0}$ for different numbers of satellites and $P_{FA}=10^{-6}$.

Figure 7 shows the spoofing validation probabilities for different numbers of satellites. The simulation parameters are the same as those in Figure 5. Since the $C/N_{0}$ of the spoofing signals are very high, $P_{V1}=1$ and $P_{V}=P_{V2}$. Results show that the probability of spoofing validation is higher when more signals are processed.

**Figure 8:**
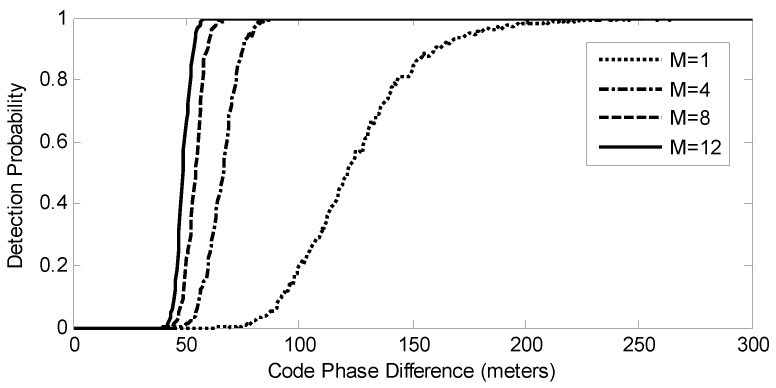
Detection probabilities versus code phase difference between spoofing and authentic signals for different numbers of satellites. $C/N_{0}$ of authentic and spoofing signals are 45 dB-Hz and 50 dB-Hz, respectively.

Then, we consider the situation when the peaks of the MLE cost function formed by spoofing and authentic signals are overlapped. Figure 8 shows the detection probabilities versus code phase difference between spoofing and authentic signals for different numbers of satellites when $C/N_{0}$ = 45 dB-Hz. The detection probability is zero when the code phases of the spoofing and authentic signals are aligned, and it increases as the code phase difference increases. When only one signal is processed, the detection probability is one only when the code phase difference is larger than 250 m, and when 12 signals are processed simultaneously, the detection probability is one as long as the code phase difference is larger than 60 m. Figure 8 demonstrates that not only more numbers of satellites, but also larger code phase differences can improve the detection performance. This improvement comes from the integration of multiple signals. The ROC curves determined by Equations (21) and (22) can be viewed as the upper bound of the detection performance. This bound can be nearly reached when the correlation peaks of spoofing and authentic signals are separated completely.

## 6. Performance Evaluation with TEXBAT

The Texas Spoofing Test Battery (TEXBAT) is a set of digital recordings of GPS L1 C/A spoofing tests. It is provided by the Radio Navigation Laboratory (RNL) at the University of Texas at Austin for evaluating civil GPS spoofing countermeasures [30].

The TEXBAT consists of eight data recordings representing different types of GNSS spoofing attacks at the time of writing. In the paper, Datasets 2 to 7 (ds2 to ds7) are employed to evaluate the proposed anti-spoofing method. They represent “static overpowered time push”, “static matched-power time push”, “static matched-power position push”, “dynamic overpowered time push”, “dynamic matched-power time push” and “seamless static matched-power time push”, respectively. The lengths of ds2 to ds6 are 400 s, and the length of ds7 is 465 s.

Ds1 is a “static switch” spoofing attack, which removes all of the authentic signals when spoofing signals appear. It cannot be detected by the MLE-based method since authentic and spoofing signals do not exist simultaneously. ds8 represents a security code estimation and replay (SCER) attack, which is identical to ds7, except that the spoofer guesses and generates the navigation data bits in real time. Since our method does not employ the security codes to detect a spoofing attack, ds8 is also not considered in the paper.

The Doppler measurements from a conventional receiver are employed in the experiment; therefore, the velocity does not need to be searched, and the search space is $\left\{x,y,z,t_{u}\right\}$. The center of the search space is set to the user position and clock offset results obtained from the conventional receiver. The search range of each dimension and the search step length of the two MLE position modules in Figure 4 are given in Table 2. Shorter search step improves the accuracy of the recovered PVT results. However, it also leads to a higher computational burden. Consequently, a trade-off needs to be made in practical applications.

Table 3 shows the spoofing detection and validation performance in Scenarios 2 to 7 of TEXBAT. *M* denotes the number of the processed signals. Here, the detection performance is measured by the spoofing detection percentage ($SDP_{i}$), which is defined as the ratio between the data length of successful detections when *i* signals are processed ($L_{i}$) and the data length under spoofing attack ($L_{S}$). The validation performance is measured by the spoofing validation percentage (SVP), which is defined as the ratio between the data length of successful validations ($L_{V}$) and $L_{S}$. The improvement of multi-signal processing is also given in Table 3, which is defined as $Imp=SDP_{M}-SDP_{1}$.

The anti-spoofing module is called every second. Therefore, 400 T(y) are calculated in each dataset of ds2 to ds6, and 465 T(y) are calculated in ds7. $L_{S}$ is about 290 in each dataset of ds2 to ds6 and 315 in ds7.

Table 3 demonstrates that in all of the scenarios, spoofing attacks can be detected. The detection performance is better when more signals are processed in each scenario. Table 3 also shows that the spoofing detection percentages are lower in ds4 and ds6, which mislead the positioning results of the victim receiver. The reason is that the code phase differences induced by the spoofing signals are smaller in these two scenarios. In addition, two groups of consistent signals are found in all of the scenarios. Thus, the detected evil signals can be judged as spoofing ones.

In particular, in order to view the performance of the proposed method intuitively, detailed spoofing detection, validation and recovered PVT results of ds2 (Static Over-Powered Time Push) and ds6 (Dynamic Matched-Power Position Push) are provided. Detection results of SQM and RAIM of the two scenarios are also provided for comparison. These two scenarios are chosen because they include different motion states of victim receivers and different power levels of spoofing signals. In addition, the spoofing attacks in the two scenarios falsify the positioning and timing results, respectively.

### 6.1. Scenario 2 of TEXBAT

**Figure 9:**
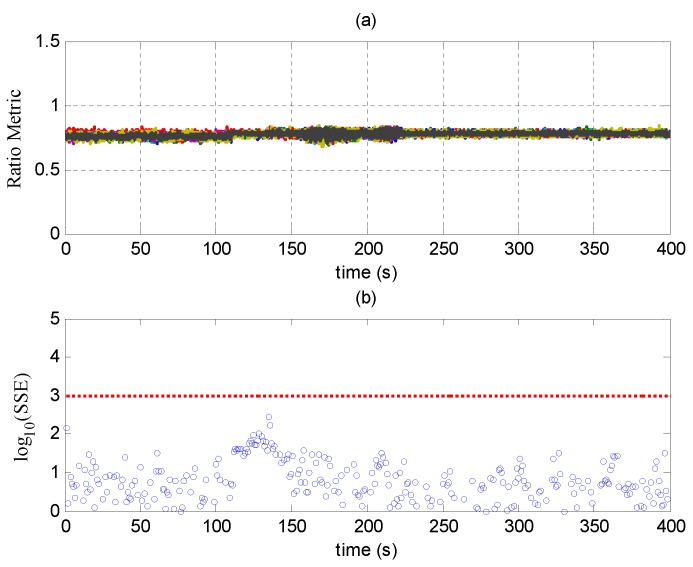
(**a**) Ratio metric of signal quality monitoring (SQM) in Scenario 2; (**b**) SSE of RAIM in Scenario 2; the red dashed line denotes the threshold.

Detection results of SQM and RAIM in Scenario 2 are shown in Figure 9. Figure 9a shows the ratio metric, which is one of the most widely-used metrics in the SQM technique. It is defined as $\left(I_{E}+I_{L}\right)/\left(2I_{P}\right)$, where $I_{E}$, $I_{L}$ and $I_{P}$ are the early, late and prompt taps on the in-phase component, respectively [15]. Here, the correlator spacing is set to 0.25 code chip; therefore, the ratio metrics should be about 0.75 when the spoofing signal is absent. It can be seen that the ratio metrics in Scenario 2 are constant throughout the experiment and fail to detect the spoofing attack. This is because the power of the spoofing signal is much higher, and no obvious disruptions occur in the drag-off process.

Figure 9b shows the sum of the squares of the range residual error (SSE), which is a statistic in RAIM technology. It is employed to evaluate the consistency of the pseudorange measurements of different signals when more than four satellites are observed at the same time [16]. When the measurements are inconsistent, the range residual error will be large, and the SSE will be over a pre-assigned threshold, implying that the PVT results are unreliable [16]. Figure 9b shows that the SSEs are all beneath the threshold and fail to detect the spoofing attack. This is because that all of the tracking channels are taken over by the spoofing signals, and the code phases of these signals are manipulated sophisticatedly to generate consistent pseudorange measurements.

**Figure 10:**
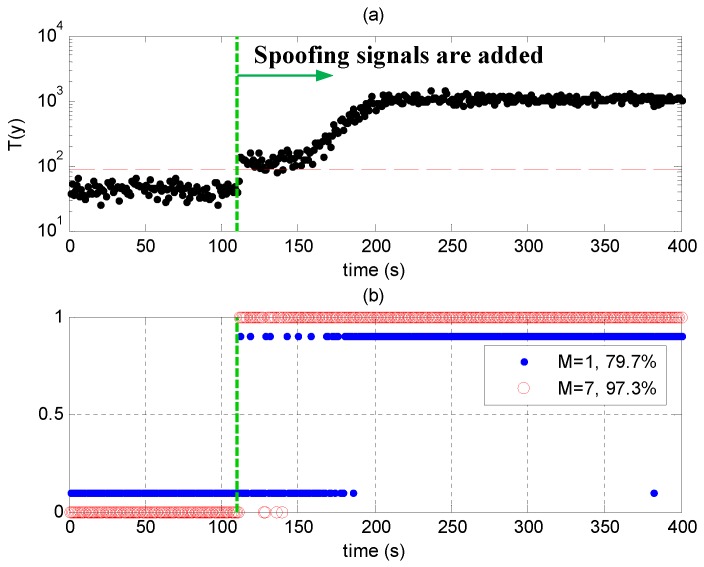
(**a**) Test statistics and threshold in Scenario 2; (**b**) Detection results in Scenario 2; results larger than 0.5 mean that there are evil signals. The red circles correspond to the results when seven signals are processed together, and the blue points correspond to the results when one signal is processed.

Figure 10a shows the test statistics T(y) in Scenario 2. They are smaller than the threshold in the first 110 s when no spoofing signals are present. At about 110 s, spoofing signals are added, and the statistics are over the threshold because the code phases of the spoofing signals are not perfectly aligned with those of the authentic ones. The statistics increase when the code phase differences between authentic and spoofing signals are larger, indicating that the spoofing signals are more likely to be detected, which is consistent with the results in Figure 8. They stop increasing after the correlation peaks of the authentic and spoofing signals are completely separate (after about 210 s).

Figure 10b shows the detection results of Scenario 2. Results larger than 0.5 mean that evil signals are detected. The red circles correspond to the results when seven signals are processed together, and the blue points correspond to the results when only one signal is processed. Figure 10b shows that the detection performance is better when more signals are processed simultaneously.

**Figure 11:**
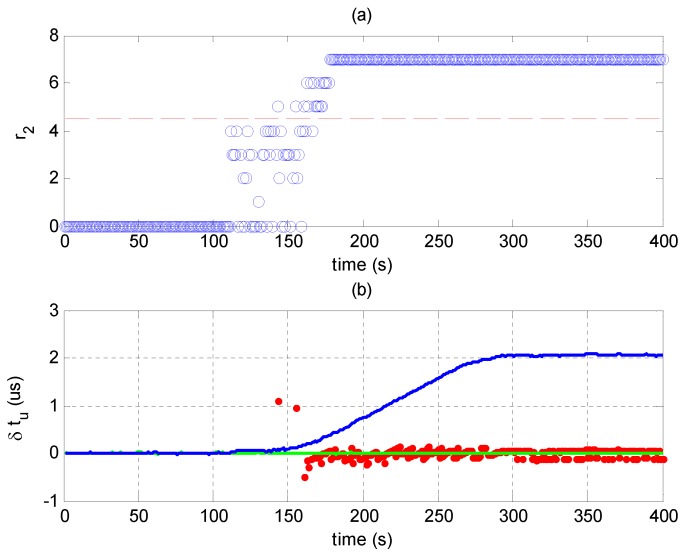
(**a**) $r_{2}$ in Scenario 2; results larger than four indicate that consistent signals are detected in the residual signal and the spoofing attack is validated; (**b**) Biases of the user clock offsets from the clock offset in Epoch 0: $\delta t_{u}=t_{u}\left(t\right)-t_{u}\left(0\right)$. The green trace shows the receiver’s unspoofed response; the blue trace shows the receiver’s spoofed response; and the red dots show the recovered results.

Figure 11a shows $r_{2}$ in Scenario 2. $r_{1}$ is omitted since it is larger than four throughout the experiment. $r_{2}$ is larger than four after 150 s, indicating that consistent signals are detected in the residual signal y. Thus, the evil signals can be judged as spoofing ones, and one of ${\hat{\rho }}_{1}$ and ${\hat{\rho }}_{2}$ corresponds to the authentic result. Here, since the total power of the spoofing signals is higher, the spoofing signals will be constructed and canceled in the estimation-cancellation procedure. Consequently, ${\hat{\rho }}_{2}$ corresponds to the authentic PVT results.

Figure 11b shows the biases of the user clock offsets in Scenario 2. The spoofed, unspoofed and recovered PVT results are indicated by blue, green and red traces, respectively. The recovered results are only shown when $r_{2}$ is larger than four. Since the spoofing signals only falsify the timing results, only the biases of the user clock offsets are shown. Figure 11b shows that the red trace (recovered result) is close to the green trace (unspoofed response) after the time error induced by spoofing signals is more than 0.2 μs (about 1/5 C/A code chip length), which demonstrates that the proposed method can recover the navigation solution even when the code phase differences between the spoofing and authentic signals are much smaller than one code chip.

### 6.2. Scenario 6 of TEXBAT

**Figure 12:**
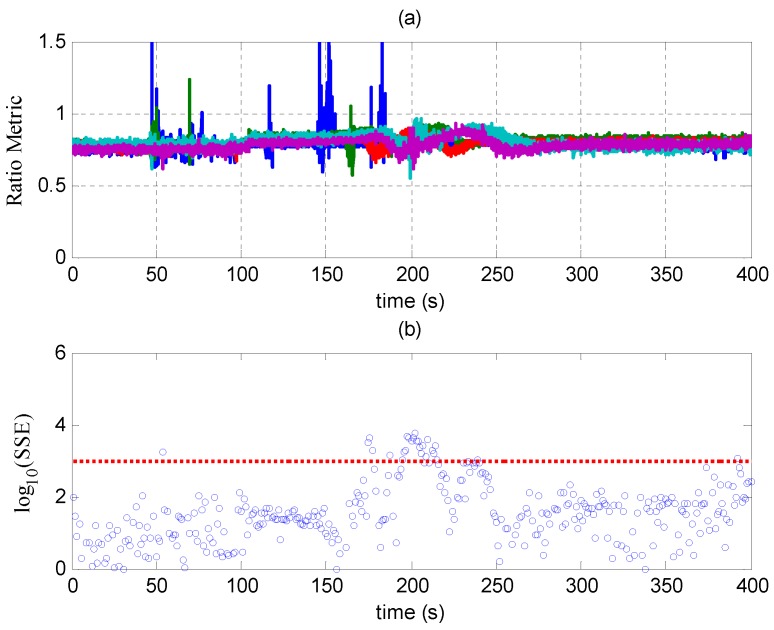
(**a**) Ratio metric of SQM in Scenario 2; (**b**) SSE of RAIM in Scenario 2; the red dashed line denotes the threshold.

Detection results of SQM and RAIM in Scenario 6 are shown in Figure 12. Different from Scenario 2, the ratio metrics and SSEs occasionally exceed the threshold. The abnormalities before 110 s are probably caused by the dynamics of the receiver. There are abnormalities between 150 and 250 s since the spoofing signals try to mislead the code phases and cause disruptions in the tracking loops of the receiver. After drag-off, both the metrics are normal again, but the receiver is completely spoofed and can only output falsified PVT results. Figure 12 demonstrates that the SQM and RAIM techniques cannot deal with the spoofing attack in Scenario 6.

**Figure 13:**
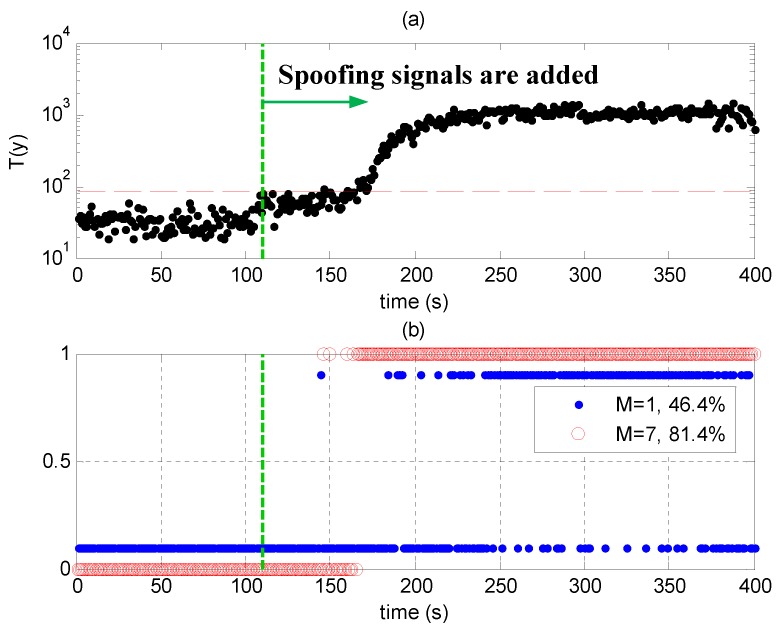
(**a**) Test statistics and threshold in Scenario 6; (**b**) Detection results in Scenario 6; results larger than 0.5 mean that there are evil signals. The red circles correspond to the results when seven signals are processed together, and the blue points correspond to the results when one signal is processed.

Figure 13a shows the test statistics in Scenario 6. They are smaller than the threshold in the first 110 s when no spoofing signals are present. From 110 s to 150 s, spoofing signals are added, but their code phases are aligned with those of the authentic ones, and their powers are only slightly higher than those of the authentic ones. Consequently, the spoofing and authentic signals will be canceled simultaneously, and the signal component in the residual signal y is very weak. Hence, the test statistics do not increase obviously. After 150 s, the test statistics increase because the code phase differences between the authentic and spoofing signals increase. Similar to Figure 10a, the statistics stop increasing after the peaks of the authentic and spoofing signals are completely separated.

Figure 13b shows the detection results of Scenario 6. It can be seen that when only one signal is processed, the detection probability in a dynamic receiver is much lower than that in a static receiver due to the motion of the receiver and the changing environment. However, the detection performance is still very good when all of the signals are processed together, since it takes advantage of the diversity gain of multiple signals.

**Figure 14:**
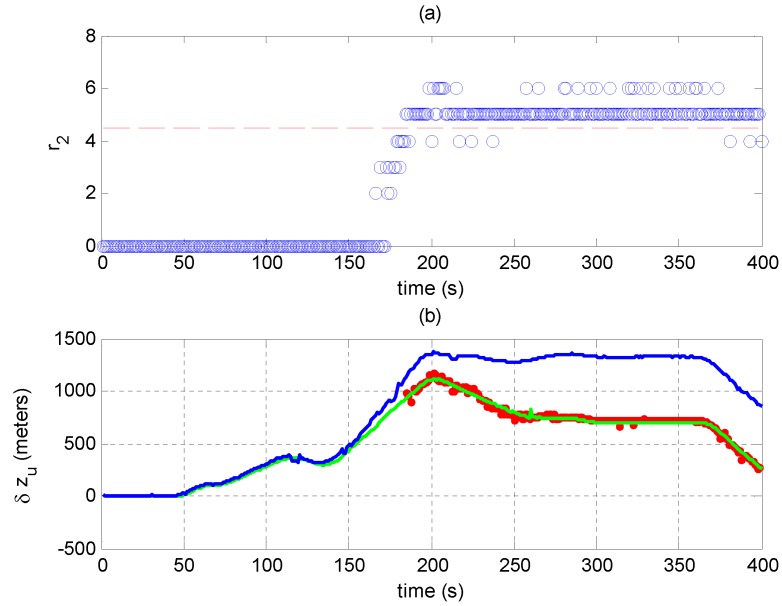
(**a**) $r_{2}$ in Scenario 6; results larger than four indicate that consistent signals are detected in the residual signals and the spoofing attack is validated; (**b**) Biases of user position in the z-coordinate from the position in Epoch 0: $\delta z_{u}=z_{u}\left(t\right)-z_{u}\left(0\right)$. The green trace shows the receiver’s unspoofed response; the blue trace shows the receiver’s spoofed response; and the red dots show the recovered results.

Figure 14a shows $r_{2}$ in Scenario 6. It is larger than four after 180 s, indicating successful spoofing validations.

Figure 14b shows the biases of user position results in Scenario 6. Since the spoofing signals in this scenario only falsify the position results in the z-coordinate, only the biases in this dimension are shown. Figure 14b shows that the recovered position results are also very close to the receiver’s unspoofed response.

## 7. Conclusions

In this paper, we develop an estimation-cancellation approach for GNSS spoofing detection and navigation solution recovery based on the MLE-based positioning technique. The proposed method distinguishes the spoofing signals from the authentic ones based on the fact that the spoofing and authentic signals form peaks at different positions of the MLE cost function. After canceling the composite signal constructed with the ML estimates of the PVT parameters, another peak can still be detected in the MLE cost function of the residual signal if evil signals such as spoofing signals are present. In order to analyze the theoretical performance and set the threshold appropriately, a test statistic is derived based on the GLRT. Furthermore, once evil signals are detected, spoofing validation can be performed by decomposing the cost function and inspecting the consistency of the signals. We also present theoretical and simulation results to investigate the anti-spoofing performance. Finally, we evaluate the proposed countermeasure with the TEXBAT GPS spoofing datasets. Results show that the method can detect and recover navigation solution even when the code phase differences between authentic and spoofing signal are much less than one code chip, which greatly improves the availability of GNSS service under spoofing attacks.

## Figures and Tables

**Table 1:** Detailed classification based on the numbers of spoofing and authentic signals in x and y.

No.	Case	*x*		${\hat{\rho }}_{1}$	*y*		${\hat{\rho }}_{2}$	*r*		Detection	Validation
		spf	auth		spf	auth		$r_{1}$	$r_{2}$		
1	Case 1	>4	>4	=$\rho_{s}$	=0	>4	=$\rho_{a}$	>4	>4	*√*	*√*
2		>4	>4	=$\rho_{s}$	=0	≤4	nd ^1^	>4	≤4	*√*	×
3		>4	≤4	=$\rho_{s}$	=0	≤4	nd	>4	≤4	*√*	×
4	Case 2	>4	>4	=$\rho_{a}$	>4	=0	=$\rho_{s}$	>4	>4	*√*	*√*
5		>4	>4	=$\rho_{a}$	≤4	=0	nd	>4	≤4	*√*	×
6		≤4	>4	=$\rho_{a}$	≤4	=0	nd	>4	≤4	*√*	×
7	Case 3	>4	≤4	≠$\rho_{s}$	≠0	≤4	nd	≤4	nd	*√*	×
8		≤4	>4	≠$\rho_{a}$	≤4	≠0	nd	≤4	nd	*√*	×
9		≤4	≤4	nd	≤4	≤4	nd	≤4	≤4	*√* ^2^	×

^1^ nd means “cannot be determined”; ^2^ when the total number of signals is 4, no signal component will be reserved in y, and the detection will fail.

**Table 2:** Search range and step of the MLE-based positioning modules.

Num	Range (m)	Step (m)
1	−50 ∼ 50	2
2	−750 ∼ 750	50

**Table 3:** Spoofing detection and validation performance in different spoofing scenarios of TEXBAT.

	*M*	${\mathrm{SDP}}_{1}$ (%)	${\mathrm{SDP}}_{M}$ (%)	Imp(%)	SVP(%)
ds2	7	79.7	97.3	17.6	82.5
ds3	6	77.3	84.9	7.6	77.3
ds4	8	64.9	78.7	13.8	61.2
ds5	6	69.4	93.5	24.1	49.0
ds6	6	46.4	81.8	35.4	71.5
ds7	7	74.7	82.0	7.3	72.2

## Abbreviations

The following abbreviations are used in this manuscript:

GNSS	Global navigation satellite system
MLE	Maximum likelihood estimation
GLRT	Generalized likelihood ratio test
PVT	Position, velocity, and time
GPS	Global Positioning System
PMU	Phasor measurement units
INS	Inertial navigation system
SSSC	Spread spectrum security code
NMA	Navigation message authentication
SQM	Signal quality monitoring
RAIM	Receiver autonomous integrity monitoring
TEXBAT	Texas Spoofing Test Battery
AWGN	Additive white Gaussian noise
ECEF	Earth-centered Earth-fixed
ROC	Receiver operating characteristic
SCER	Security code estimation and replay
